# Multiresponse Surface Optimization of Ionic Gelation Vibrational Jet Flow Technology to Fine‐Tune Kafirin Microparticles Extracted From Sorghum Dried Distiller's Grain

**DOI:** 10.1111/1750-3841.70268

**Published:** 2025-05-22

**Authors:** Umar Shah, Rewati R. Bhattarai, Hani Al Salami, Chris Blanchard, Stuart K. Johnson

**Affiliations:** ^1^ School of Molecular and Life Sciences, Faculty of Science and Engineering Curtin University Perth Western Australia Australia; ^2^ The Biotechnology and Drug Development Research Laboratory, Curtin Medical School and Curtin Health Innovation Research Institute Curtin University Perth Western Australia Australia; ^3^ ARC ITTC for Functional Grains, Graham Centre for Agricultural Innovation Charles Sturt University Wagga Wagga New South Wales Australia

**Keywords:** DDGS kafirin, ionic gelation vibrational jet flow technology, microparticles, response surface methodology

## Abstract

Sorghum dried distiller's grain with solubles (DDGS), a protein‐enriched byproduct of sorghum bioethanol production, is predominantly used as a low‐cost animal feed. However, unutilized DDGS is mainly discarded as waste, containing approximately 40% of the prolamin protein kafirin. Kafirin's high hydrophobicity, low digestibility, evaporation‐induced self‐assembly, and strong disulfide cross‐linking offer potential for biomaterial applications. This study used ethanol extraction and acid precipitation to purify kafirin protein from sorghum DDGS. The extracted protein was then used to prepare microparticles using ionic gelation vibrational jet flow technology (IGVJFT). This technology enables reproducible, uniform, scalable, high‐speed microparticle production compared to existing methodologies. The integrated electrode voltage (V), internal frequency/vibration (Hz), and DDGS kafirin concentration (% [w/v]) used in the IGVJFT process were evaluated against key microparticle physicochemical response factors of volume‐weighted mean microparticle size (% [w/v]), zeta potential (mV), and fracture frequency (mechanical strength) (%). Optimization of microparticle formation was performed by a response surface methodology (RSM) central composite design. Under different processing parameters of the RSM, the resulting DDGS kafirin microparticles possessed spherical morphology, volume‐weighted mean particle sizes from 406.7 to 656.4 µm, zeta potential in the range of −38.2 to −18.1 mV, and fracture frequency (mechanical strength) of 23%–57%. Optimal conditions for producing microparticles with minimal size, high negative zeta potential, and low fracture frequency were identified and validated. These findings highlight the potential of DDGS kafirin as a sustainable material for large‐scale microparticle applications and demonstrate the efficacy of IGVJFT for assembling hydrophobic proteins into microparticles with tailored physicochemical properties.

## 1 Introduction

Grain‐based ethanol production, such as from maize and sorghum, has received adequate attention in the last decade because of environmental and sustainable economies (Rosenboom et al. 2022; Shah et al. 2024). This industry leaves a value‐added byproduct called dried distiller's grain with solubles (DDGS) (Shah et al. 2021). The increased attention toward DDGS as a biomaterial is due to its high prolamin protein content, which contains around 50% hydrophobic amino acids and is slowly digestible (Ye et al. 2024). At present, this protein‐rich byproduct, DDGS, is used as low‐cost animal feed, and a large amount of this goes to waste. These hydrophobic prolamin proteins, such as kafirin from sorghum and zein from maize, are not suitable for direct human consumption because of their negative nitrogen balance and poor solubility in water (Semwal and Meera 2024; Shukla and Cheryan 2001). It is, therefore, essential to identify alternative uses for these proteins: one such potential application is based on their unusual physicochemical properties for the production of biomaterials.

Sorghum kafirin has triggered a new wave in biomaterial research owing to its unique physicochemical properties related to material behavior (Lau et al. 2015; Taylor and Taylor 2018). To date, most of the research on kafirin material use, such as delivery agents, is based on kafirin purified from sorghum grain (Xiao et al. 2015). The interesting material behavior of kafirin is due to its unique functionalities, such as a high ratio of hydrophobic amino acids to hydrophilic amino acids, high content of disulfide cross‐linking, high hydrophobicity, and slow digestibility behavior (Belton et al. 2006; Duodu et al. 2003). Of particular interest is the kafirin protein within sorghum DDGS, which can be recycled and utilized for value‐added commercial use (Shah et al. 2021; Wang et al. 2009). As such, globally, authorities have identified priorities for the value‐added utilization of DDGS (Chatzifragkou et al. 2015).

We have analyzed the physicochemical properties of kafirin extracted from sorghum DDGS for material application and compared them to those of kafirin obtained from the original sorghum grain (Shah et al. 2021). Both kafirins demonstrated comparable primary, secondary, tertiary, and quaternary structures as well as some similarities in morphological properties, which indicates that DDGS kafirins are a suitable alternative to that extracted from the grain for biomaterial applications. Of interest, a small variation in protein folding between kafirin from DDGS and that from the grain was observed by Fourier transform infrared spectroscopy (FTIR). This indicated a minor difference in secondary structure, specifically the unfolding of some α‐helices and random coils, followed by realignment and reorganization into β‐sheets. The higher β‐sheets content in DDGS kafirin than grain kafirin suggests its suitability for a viscoelastic self‐assembly delivery system. Microparticles with viscoelastic properties are known for their high energy absorption, shock resistance, and damping behavior. Additionally, morphological analysis of the DDGS kafirin showed the formation of less spherical particles with internal pores, in contrast to grain kafirin, which formed spherical‐shaped particles with homogeneous surfaces made using an evaporation‐induced self‐assembly mechanism.

Technologically, processes such as antisolvent precipitation (Hu et al. 2022), co‐precipitation (Sun et al. 2025), pH cycling (Sincari et al. 2024), and solvent evaporation (Vladisavljević 2024) have been developed for the preparation of microparticles. Each of these approaches, however, has drawbacks, such as limited commercial applicability due to high solvent usage and challenges in controlling the physicochemical properties of the resulting particles (Song et al. 2021). Similarly, processes such as spray drying (Lee et al. 2010), ultrasonication (Liu et al. 2016), freeze‐drying (Quispe‐Condori et al. 2011), and electrospinning (Xu et al. 2017) have been developed for the production of hydrophobic protein microparticles; however, each approach has drawbacks. For example, spray drying causes polymorphic transformations and sticking of material to chamber walls, as well as involves the use of heat, which is not suitable for proteins. Ultrasonication causes cavitation, which may lead to free radical formation (Leong et al. 2017). Electrospinning produces nonuniform fibers, while removing residual solvent and handling the final fibers are difficult (Davila et al. 2021; Rostamabadi et al. 2020). Recently, advanced technologies have been developed for the preparation of spheroids and organoids (Guazzelli et al. 2023).

One novel approach for microparticle formation from biopolymers is ionic gelation vibrational jet flow technology (IGVJFT). This technique is known to produce particles with high reproducibility by integrating mechanical (i.e., vibrational nozzle for particle sizing) and chemical (i.e., ionic gelation for particle curing) processes (Kovacevic et al. 2024; Wagle et al. 2020). Advantages of IGVJFT over traditional microparticle production methods include the use of less energy; particle size uniformity; controllable process with visual monitoring; and high‐speed production (Jones et al. 2020; Mooranian et al. 2020; Wagle et al. 2020). The key components of this technology are a dispensing bottle, a nitrogen‐based airflow system, a particle production unit that contains a nozzle electrode with controlled voltage, frequency, and nozzle size, an airflow pump, a stroboscope, and a polymerization bath (e.g., calcium chloride dissolved in water) with a magnetic stirrer. The biopolymer solution in the dispensing bottle is forced into the particle production unit by the airflow system, where it passes through a nozzle, and with the aid of vibrational frequency and integrated voltage, equal‐sized droplets are created. These are sprayed from the nozzle into a polymerization bath that induces their solidification. Only a limited number of studies have reported on the manufacture of microparticles using IGVJFT. For instance, this technology has been used to make microparticles from poly‐l‐ornithine, sodium alginate, and ursodeoxycholic acid to encapsulate various drugs and cells (Wagle et al. 2024). The authors reported that IGVJFT produced stable microparticles with high encapsulation efficiency.

Laboratory‐scale new technologies, such as IGVJFT, require optimization before they can be scaled up. One commonly used experimental design for such optimization is response surface methodology (RSM). To date, no studies have utilized high‐speed IGVJFT for the production of prolamin protein microparticles. Additionally, there is a research gap in the fabrication of industrial waste‐based biomaterial using this technology, specifically those soluble in binary solvents, such as alcohol–water mixture used for DDGS kafirin. The aim of the current study was to optimize the production process of IGVJFT for the preparation of DDGS kafirin microparticles. A central composite design (CCPD) coupled with a desirability function was employed. This study will develop optimal conditions for the production of DDGS kafirin microparticles with controllable size, shape, stability, and fracture frequency.

## 2 Material and Methods

### 2.1 Materials

Sodium hydroxide, sodium metabisulphite, *n*‐hexane, methanol, and calcium chloride were purchased from Sigma–Aldrich (Castle‐Hill, NSW, Australia). Absolute ethanol and HCl were obtained from Thermo‐Fisher Scientific (Scoresby, VIC, Australia). The water used in this research was purified by Ultrapure Technology (Life Technologies, USA). The conductivity of the purified water was 0.055 µS/cm.

### 2.2 Extraction of Kafirin Protein

Sorghum DDGS (10 kg) was gifted by Balby Bio‐Refinery (Dalby, Queensland, Australia). It was vacuum packed and stored at 4°C before further use.

The extraction procedure used in this research is based on that of Shah et al. (2021). Sorghum DDGS was ground using a pin mill (Cemotec 1090 sample mill; Foss Tecator, Mulgrave, VIC, Australia) and blended (ZM 200 blender, Retcsh GmbH & Co, Haan, Germany), followed by sieving through a 500‐micron sieve, yielding 95% recovery. For extraction, 50 g of milled DDGS was soaked in 250 mL of an extraction solution containing 62% ethanol, 0.064% NaOH, and 0.22% Na_2_S_2_O_5_, and incubated (Memmert 854, Schwabach, Germany) at 60°C with agitation (150 rpm) for 1 h. After cooling to 25°C, the mixture was sonicated (30 Hz, 60 W; Ultrasonic cleaner, DSA, Madrid, Spain) for 5 min, centrifuged (1750 × *g*, 20 min, 4°C; Eppendorf Centrifuge 5810 R, Macquarie Park, NSW, Australia), and the supernatant was concentrated via vacuum rotary evaporation. Kafirin was precipitated by adjusting pH to 5.0, allowed to stand overnight, centrifuged, and dried at 40°C. The dried pellet was defatted using three hexane washes, followed by evaporation at 60°C, and then blended. Particle size analysis using Master Sizer 2000 (Malvern Instruments Ltd, Malvern, UK) (Zhong et al. 2019) determined a volume‐weighted mean particle size of 272 µm. The protein content was 84.76 ± 0.76 g/100 g dry basis (db) measured using elemental analysis (2400, Perkin Elmer Pvt Ltd, Macquarie Park, NSW, Australia). The extraction yield was ∼59% (g protein extract [db]/g DDGS [db] × 100) (Shah et al. 2021).

### 2.3 Experimental Design

#### 2.3.1 Modeling Procedure of RSM

A response surface CCPD, with 20 runs, was produced using Design Expert V11 software (Stat‐Ease Inc., Minneapolis, MN, USA). The CCPD had 20 production runs in total, each with different levels of dependent process parameters (Table 1). These parameters were a short‐list selected as having the most significant effect on the microparticle attributes in a preliminary study using factorial screening.

**TABLE 1:** Independent process parameters of ionic gelation vibrational jet flow technology.

Factor	Process parameters	Unit	−α	−1	0	+1	+α
K	DDGS kafirin concentration	% (w/v)	0.8	1	1.5	2	2.15
V	Integrated electrode voltage	V	136	200	400	600	663
F	Internal frequency/vibration	Hz	789	900	1250	1600	1710

*Note*: −α denotes a low value; −1 denotes the minimum value; 0 denotes multiple central points; +1 denotes the maximum value; and +α denotes a high value.

A generalized polynomial quadratic regression equation was used to provide detailed insights into how the process parameters, DDGS kafirin concentration (*K*), internal electrode voltage tension (*V*), and internal vibration/frequency (*F*), affected the selected dependent process parameters, volume‐weighted mean microparticle size (µm), zeta potential (mV), and fracture frequency (%).

In Equation (1), the equation of regression, *Y* represents the response, *β*_0_ is the intercept, *β_i_*, *β_ii_*, and *β_ij_* are regression coefficients, and $X_{i},X_{j}$ represent the independent variables at *i* and *j* levels. The CCPD design runs are represented in Table 2, where the center points were repeated to provide an assessment of experimental error.

(1) $$Y=\beta_{0}+\sum_{i=1}^{n}\beta_{i}X_{i}+\sum_{i=1}^{n}\beta_{ii}X_{i}^{2}+\sum \sum_{i<j=2}^{n}\beta_{ij}X_{i}X_{j}.$$

**TABLE 2:** Experimental levels of process parameters of ionic gelation vibrational jet flow technology and response results as given by the central composite design of response surface methodology (RSM).

		Factors			Responses		
Run	*K* (% [w/v])	*V* (V)	*F* (Hz)	PS (µm)	ZP (mV)	FF (%)	SEM
1	2.15	400	1250	618 ± 34	−14.7 ± 1.5	21 ± 3	Figure 4A
2	1	600	900	470 ± 22	−33.1 ± 4.6	48 ± 7	
3	1.5	400	1250	562 ± 32	−24.5 ± 3.3	39 ± 6	
4	2	600	900	656 ± 35	−18.1 ± 5.1	23 ± 2	
5	1.5	400	1250	585 ± 28	−28.9 ± 3.2	37 ± 4	Figure 4B
6	1	200	1600	449 ± 27	− 35.8 ± 1.3	51 ± 1	
7	2	600	1600	554 ± 17	−22.4 ± 0.6	32 ± 6	
8	2	200	900	624 ± 34	−16.9 ± 1.9	18 ± 7	
9	2	200	1600	560 ± 42	−20.3 ± 2.6	24 ± 2	Figure 4C
10	1.5	136.7^*^	1250	623 ± 52	−24.6 ± 3.2	33 ± 8	
11	1.5	400	789.3^*^	605 ± 25	−25.4 ± 5.2	32 ± 6	
12	1	600	1600	406 ± 14	−38.2 ± 3.2	57 ± 12	Figure 4D
13	1.5	663.2^*^	1250	540 ± 19	−29.9 ± 3.9	44 ± 6	
14	1.5	400	1710.6^*^	451 ± 38	−31.1 ± 6.1	35 ± 13	
15	1.5	400	1250	639 ± 28	−25.9 ± 1.9	39 ± 12	
16	0.8	400	1250	457 ± 14	−30.1 ± 3.1	47 ± 7	Figure 4E
17	1	200	900	523 ± 34	−29.6 ± 5.3	45 ± 4	
18	1.5	400	1250	557 ± 38	−26.5 ± 3.3	41 ±6.7	
19	1.5	400	1250	567 ± 22	−27.2 ± 3.6	36 ± 2	
20	1.5	400	1250	560 ± 21	−27.9 ± 4.3	39 ± 8	Figure 4F

*Note: K* = concentration of DDGS kafirin; *V* = integrated electrode voltage; *F* = internal frequency; PS = volume‐weighted mean DDGS kafirin microparticle size; ZP = zeta potential of DDGS kafirin microparticles; FF = fracture frequency of DDGS kafirin microparticles; SEM = scanning electron microscopy of selected model runs. * rounded to nearest possible decimal.

#### 2.3.2 Simulations Using the Desirability Function

Simulations were performed on the CCPD response data to identify optimal levels of all three processing factors in combination, predicted to give the highest quality microparticles with a small volume‐weighted mean particle size, high negative zeta potential, and high fracture frequency. A global desirability function (Equation 2) (Vera Candioti et al. 2014) was applied to the data:

(2) $$D=\left(d_{1}^{r_{1}}.d_{2}^{r_{2}}.\cdots d_{n}^{r_{n}}\right)\frac{1}{\sum r_{i}}={\left(\prod_{i=1}^{n}d_{i}^{r_{1}}\right)}^{\frac{1}{\sum r_{i}}}$$

where *d*_1_, … *d_n_* is the desirability function, *n* represents the number of responses, and *r_i_* is the degree of priority criteria score.

#### 2.3.3 Model Validation

To validate the CCPD model, point prediction confirmation of an optimal statistical solution and a suboptimal solution was performed (Villarino et al. 2015). The microparticle production was conducted in duplicate, utilizing (a) optimal levels of process parameters that achieved the target levels of the microparticle independent variables as predicted by the desirability function (see Section 2.3.2) and (b) levels of process parameters predicted to achieve suboptimal levels of the microparticle independent variables. Experimental data for each response parameter were compared to the predicted response value using confidence and prediction intervals at *α* = 0.95. When experimental values of the responses are within the confidence and/or prediction interval, the ability of the model to accurately predict responses is validated.

### 2.4 IGVJFT

Feed solutions were formulated according to the concentrations of DDGS kafirin given by the RSM CCPD experimental design (see Section 2.3.1) (Table 1). IGVJFT was operated in batch mode. The liquid flow rate of 4 mL/min, gauge air pressure of 300 mbar, and nozzle diameter of 300 µm were used for all runs, as the preliminary factorial screening of a wider range of processing parameters indicated that they had less impact on the microparticle independent variables (Shah et al. submitted) and would form discreet microparticles.

### 2.5 Determination of DDGS Kafirin Microparticle Size (PS)

The volume‐weighted mean size of microparticles (µm) was measured using a Mastersizer 2000 (Malvern Instruments Ltd, Worcestershire, UK). The dried microparticles were suspended in purified water (Life Technologies, USA) before analysis (Lau et al. 2015). Each analysis was performed in triplicate.

### 2.6 Determination of Zeta Potential of DDGS Kafirin Microparticle (ZP)

The zeta potential (mV) of the microparticles was determined by a Zetasizer 3000HS (Nano S, Malvern Instruments, Worcestershire, UK). The measurements were performed by suspending 0.01% (w/v) of microparticles in 0.5 mL of purified water (Life Technologies, USA) and were allowed to equilibrate for 1 h under ambient conditions before analysis. The Omni SEC‐Zetasizer software package (Zeta v7.11, Malvern Instruments, Worcestershire, UK) was used to correlate the generated data to the *Z*‐average mean. The samples were analyzed in triplicate.

### 2.7 Determination of Fracture Frequency of DDGS Kafirin Microparticles (FF)

Fracture frequency (%) as a measure of the mechanical strength of the DDGS kafirin microparticles was analyzed by placing 50 microparticles in a Boeco Multishaker PSU 20 (Boeco Company, Hamburg, Germany) under shaking conditions for 30 min. The fracture frequency was calculated as in Equation (3):

(3) $$\mathrm{FF}=\frac{\mathrm{FC}}{\mathrm{TC}}\times 100,$$

where FF is the fracture frequency after 30 min of shaking; FC is the number of fractured DDGS kafirin microparticles; and TC is the total number of DDGS kafirin microparticles. Each sample was analyzed in triplicate.

### 2.8 Field Emission‐Scanning Electron Microscopy (FE‐SEM)

The surface morphology of microparticles was investigated using secondary electron (SE) imaging on a dual‐beam field emission‐scanning electron microscope (Zeiss Neon 40EsB FEBSEM, Oberkochen, Germany). Samples were kept in a desiccator, then placed onto aluminum stubs using carbon tape, and coated with 6‐nm platinum using a splutter coater (208HR, Cressington, Watford, UK). A 5‐kV electron beam was used (Liu et al. 2016).

### 2.9 Statistical Data Analysis

The model was generated and analyzed using Design‐Expert software (V11, Stat‐Ease Inc., Minneapolis, MN, USA). The algorithm that showed higher precision in terms of sequential *F*‐tests (significance of process parameters), insignificant lack of fit, *R* ^2^ (percentage of variation), and adjusted *R* ^2^ (model statistics as per process parameters) was chosen (refer to Figure S1–S5 for complete diagnostic analysis of RSM). The results of the macroparticle analyses are reported as mean ± SD (*n* = 3).

## 3 Results and Discussion

### 3.1 Modeling Volume‐Weighted Mean Microparticle Size (PS)

The microparticle volume‐weighted mean size (PS) ranged from 406.7 to 656.4 µm (Table 2). The best‐fitting model (Table 3) indicated that DDGS kafirin concentration *K* (*p* < 0.0001), internal frequency *F* (*p* < 0.0001), integrated voltage *V* (*p* = 0.0218), the interactive effect of DDGS kafirin concentration and internal voltage *K* × *V* (*p* = 0.0439), and the quadratic effect of *K* (*p* = 0.0102) and *F* (*p* = 0.0104) were significant process parameters affecting volume‐weighted mean microparticle size (Table 4). The interaction of *K* × *F* (*p* < 0.4201) and *V* × *F* (*p* < 0.5985) was insignificant and not included in the model (Frank 1992). Figure S4 shows that residuals for volume‐weighted mean microparticle size were distributed in the narrow range, which indicates that the model was suitable for predicting the response.

**TABLE 3:** Analysis of variance (ANOVA) of quadratic model for experimental responses: Volume‐weighted mean microparticle size (PS; µm), zeta potential (ZP; mV), and fracture frequency (FF; %).

Response	PS (µm)		ZP (mV)		FF (%)		Validation ANOVA
Source	*F*‐value	*P‐*value	*F*‐value	*P‐*value	*F‐*value	*P‐*value	
Model	22.52	< 0.0001	45.18	<0.0001	49.82	<0.0001	Significant
*K*	109.14	< 0.0001	308.50	<0.0001	335.44	<0.0001	
*V*	7.36	0.0218	9.10	0.0130	27.45	0.0004	
*F*	49.48	< 0.0001	26.55	0.0004	25.40	0.0005	
KV	5.31	0.0439	—	—	10.80	0.0082	
KF	—	—	—	—	12.59	0.0053	
VF	—	—	—	—	—	—	
*K* ^2^	9.97	0.0102	58.25	<0.0001	27.19	0.0004	
*V* ^2^	—	—	—	—	—	—	
*F* ^2^	14.52	0.0104	5.16	0.0465	—	<0.0001	
Lack of fit^a^							Nonsignificant^b^
Fit summary							
*R* ^2^	0.9530		0.9760		0.9782		
Adjusted *R* ^2^	0.9107		0.9544		0.9586		
Predicted *R* ^2^	0.8218		0.8927		0.8597		
Adeq. precision	17.452		24.216		24.232		
Transformation (*k* > 5), alpha = 1.49535							

*Note*: PS is the volume‐weighted mean particle size, ZP is the zeta potential, and FF is the fracture frequency of microparticles. The *F*‐value is the significance of the parameters, and the *p*‐value is the probability value.

^a^ Lack of fit denoted diagnostics like how well the full model has fitted experimental data.

^b^ Nonsignificant lack of fit means that model diagnostics are well‐fitted with the data.

**TABLE 4:** Polynomial quadratic equation fitted to values of volume‐weighted mean DDGS kafirin microparticle size, zeta potential, and fracture frequency of DDGS kafirin microparticles as a function of DDGS kafirin concentration (% [w/v]), integrated electrode voltage (V), and internal frequency (Hz).

Parameter	PS (µm)	ZP (mV)	FF (%)
*K*	−0.001177	+0.044540	+0.001412
*V*	+1.15002	−0.000018	+0.000027
*F*	−1.71540	−0.000041	−0.000020
KV	−6.33230	—	−0.000023
KF	—	—	—
VF	—	—	−0.000014
*K* ^2^	+ 0.000372	−0.026454	+0.015490
*V* ^2^	—	—	—
*F* ^2^	+9.15697	+1.60611	+1.27406

*Note: K* = concentration of DDGS kafirin; *V* = integrated voltage; *F* = internal frequency; KV = cross‐interaction between DDGS kafirin and integrated voltage; KF = cross‐interaction between DDGS kafirin concentration and internal frequency; VF = cross‐interaction between integrated voltage and internal frequency; *K* ^2^, *V* ^2^, and *F* ^2^ = quadratic effects of concentration, voltage, and frequency, respectively.

This cross‐parameter interaction between *K* × *V*, which resulted in a smaller DDGS kafirin microparticle size, is based on particle jamming (Han et al. 2025; Patel et al. 2019; Verma and Daya 2017). This means the reaction rate is governed by the concentration of DDGS kafirin, which in turn requires a voltage to overcome surface tension (Lin and Timasheff 1996; Vossoughi and Matthew 2018). The results indicate that as DDGS kafirin concentration decreased, applying a higher integrated electrode voltage facilitated the mass transfer and assembly of DDGS kafirin microparticles.

**FIGURE 1:**
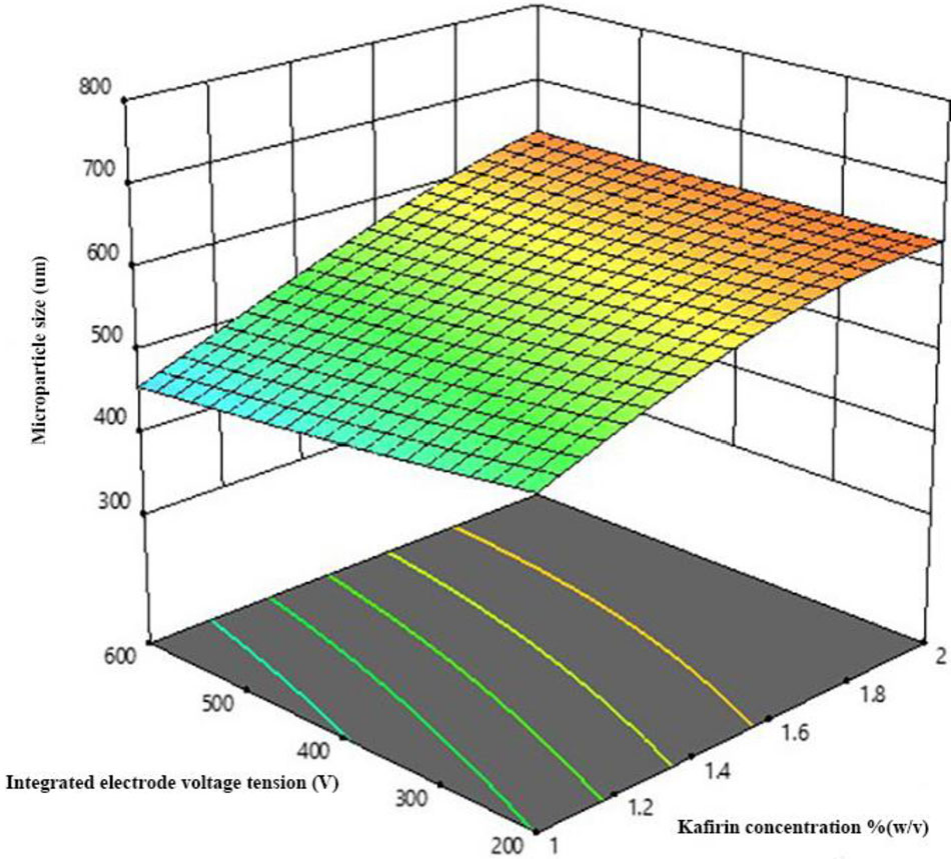
A 3D‐response surface fitted to volume‐weighted mean DDGS kafirin microparticle size as a function of kafirin concentration, *K* (% [w/v]), and integrated electrode voltage, *V* (V).

These 3D response surface diagrams of the data illustrate that a decrease in kafirin concentration combined with an increase in integrated voltage resulted in smaller DDGS kafirin microparticles (Figure 1). Higher concentrations of kafirin increase the microparticle size, which might be attributed to kafirin's higher tendency to aggregate in the gelation bath as a result of increased molecular collisions. A more viscous DDGS kafirin at higher kafirin concentrations likely requires higher voltage to overcome surface tension due to increased electrostatic forces, thus resulting in larger DDGS kafirin microparticles. As a result, a lower concentration of DDGS kafirin in the mixture led to more stable DDGS kafirin microdroplets after being ejected from the nozzle, and a higher integrated voltage helped with the solidification process. This finding is consistent with those of Liu et al. (2016), who assembled microparticles using the prolamin protein zein by ultrasonic‐assisted dialysis technology. The concentration of zein and the system energy (sonication power) significantly influenced the particle size. For example, low zein concentration (5 mg/mL) and high ultrasonic power (125 W) fabricated smaller particles with a mean size of 431.2 ± 41.3 nm. In contrast, 20 mg/mL of zein with low ultrasound power (25 W) produced particles with a larger mean size of 1809 ± 291.9 nm.

### 3.2 Modeling Zeta Potential of DDGS Kafirin Microparticles

The zeta potential of DDGS kafirin microparticles was in the range of −38.2 to −14.7 mV (Table 2). The best‐fitting model (Table 3) indicated that DDGS kafirin concentration *K* (*p* < 0.0001), integrated voltage *V* (*p* = 0.013), internal frequency *F* (*p* = 0.0004), and the quadratic effect of *K* (*p*<0.0001) and *F* (*p* = 0.0465) were significant process parameters affecting the zeta potential of fabricated microparticles (Table 4). The results indicate a less negative zeta potential as DDGS kafirin concentration increases in the feed solution. This less negative zeta potential is attributed to greater counterions, for example, DDGS kafirin concentration of 2% (w/v) when compared to 1% (w/v) (Kesimer and Gupta 2015; Mushtaq et al. 2025). The influence of internal frequency can be related to gel droplet density, that is, the lower the gel droplet density, the higher the stability. The effect of integrated voltage is based on a strong electric field that might have increased potential energy. The lower DDGS kafirin concentration resulted in greater ionic repulsion forces (higher negative zeta potential) (Nazir et al. 2024). The stability of DDGS kafirin colloidal suspension is directly proportional to the charged species present on the surface of the particle. The more negative the charge, the less chance it has to aggregate, leading to particle collapse (Kour et al. 2024). The stability of the DDGS kafirin protein‐based system is a balance between attractive and repulsive forces (Lai et al. 2016; Lowry et al. 2016); that is, the DDGS kafirin colloidal system will be more stable when repulsive forces (higher negative) are stronger than attractive forces. It is evident from the study that the concentration of DDGS kafirin is the main parameter that influences the zeta potential of fabricated microparticles. Such results align with Henry's equation, suggesting that particle velocity depends on the concentration, which determines the system's electric field strength (Hill et al. 2003).

### 3.3 Modeling Fracture Frequency of DDGS Kafirin Microparticles

The fracture frequency (an indication of mechanical strength), FF, of DDGS kafirin microparticles was in the range of 18 ± 7% to 57 ± 12% (Table 2). The best‐fitted model (Table 3) indicated that DDGS kafirin concentration *K* (*p* < 0.0001), integrated voltage *V* (*p* = 0.0004), internal frequency *F* (*p* = 0.0005), the interactive effect of DDGS kafirin concentration and internal voltage *K* × *V* (*p* = 0.0082) and DDGS kafirin concentration and internal frequency *K* ×F (*p* = 0.0053), and the quadratic effect of *K* (*p* = 0.0004) and *F* (*p* < 0.0001) were significant process parameters affecting the fracture frequency of DDGS kafirin microparticles. The results indicated that DDGS kafirin concentration has a major role in the higher mechanical strength of DDGS kafirin microparticles. The increased DDGS kafirin concentration in the stock solution may have resulted in a stronger molecular network (Shah et al. 2024; Taylor et al. 2009b; Xiao et al. 2017), which could be the reason for the higher mechanical strength. Kafirin protein, specifically β‐ and γ‐kafirins, contains numerous cysteine residues that can form disulfide bonds. As the concentration of kafirin in the stock solution increases, the frequency of intermolecular interactions, such as hydrogen bonds and disulfide bridges, increases, which contributes to the formation of a more cohesive and stable structure (Belton et al. 2006). Therefore, increasing the concentration of kafirin in the stock solution enhances the mechanical strength of DDGS kafirin microparticles by promoting a denser network of protein molecules.

The analysis of variance signifies the effect of kafirin concentration and integrated electrode voltage (*p* < 0.0001), together with a quadratic effect of internal frequency (*p* < 0.0001), on the fracture frequency of DDGS kafirin microparticles (Table 5). The fracture frequency of DDGS kafirin microparticles was also influenced by some cross‐parameter interactions, notably the effects of DDGS kafirin concentration and integrated voltage *K* × V and integrated voltage and internal frequency *V* × *F*. The coefficient of the predictive equation is tabled in Table 4.

**FIGURE 2:**
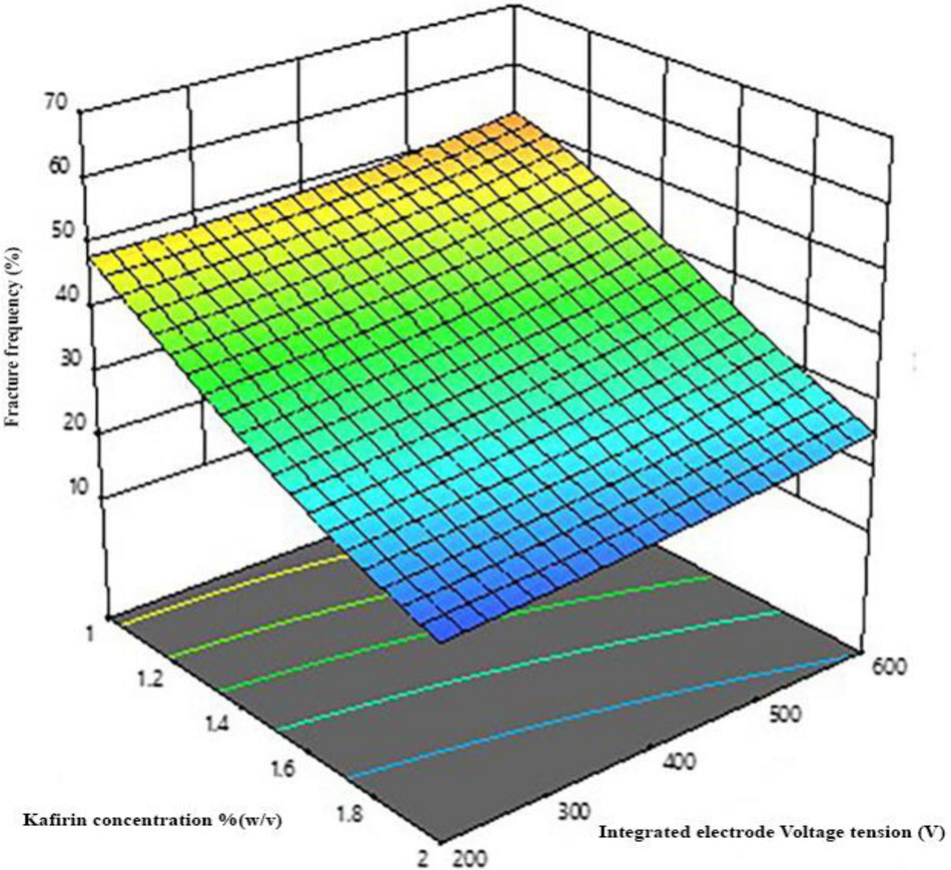
A 3D‐response surface fitted to the fracture frequency, FF (%), as a function of DDGS kafirin concentration, *K* (% [w/v]), and integrated electrode voltage, *V* (V).

**FIGURE 3:**
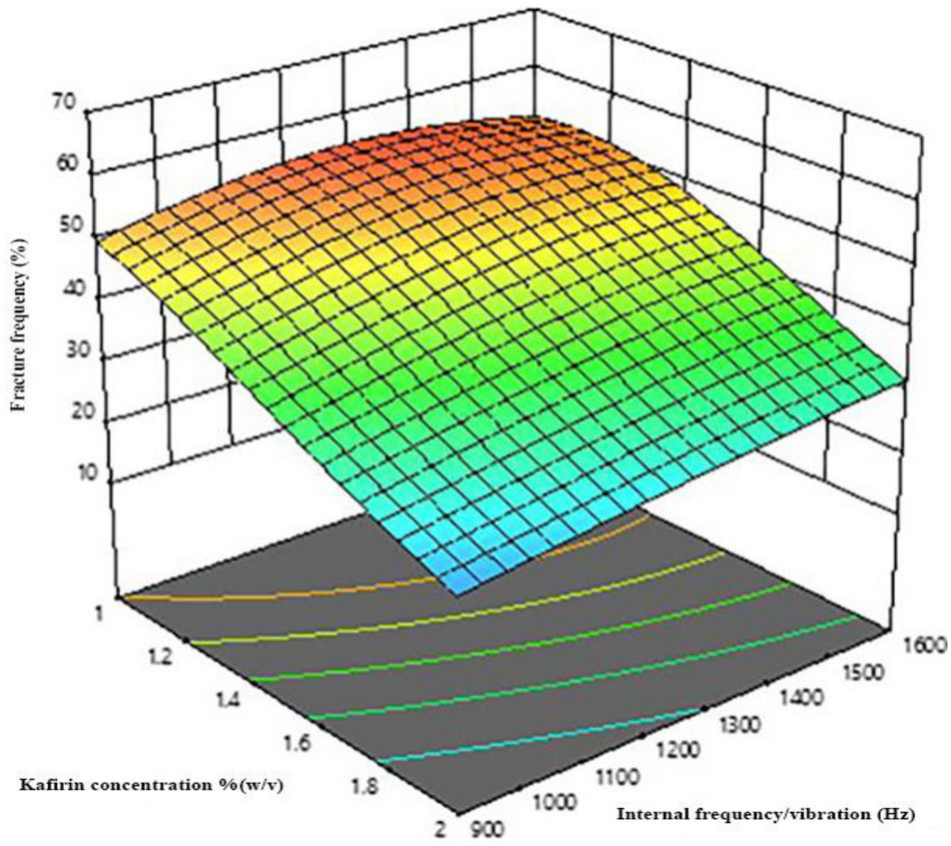
A 3D‐response surface fitted to the fracture frequency, FF (%), as a function of DDGS kafirin concentration, *K* (% [w/v]), and internal frequency, *F* (Hz).

The 3D surface plot fitted to FF as a function of the concentration of kafirin and integrated electrode voltage is shown in Figure 2. This 3D‐fitted plot suggests that the increasing concentration of kafirin (*K*; % decreased integrated electrode voltage [*V*]) resulted in the least breakage of DDGS kafirin microparticles. This interactive effect of DDGS kafirin concentration and electrode voltage may be attributed to the more intense electrostatic interactions at higher concentrations of DDGS kafirin compared to lower concentrations. At higher DDGS kafirin concentrations, the increased number of protein molecules leads to a higher charge density, which might enhance electrostatic interactions between molecules when an electrode voltage is applied. These stronger electrostatic forces, coupled with the electric field, promote better alignment and aggregation of proteins, resulting in more stable and mechanically stronger microparticles. Similar findings have previously been reported, where microparticles formulated with higher concentrations of alginate, poly‐l‐ornithine, polystyrene sulfonate, and lipophilic bile acid—produced using IGVJFT—exhibited higher fracture frequency (Mooranian et al. 2018). Figure 3 shows a 3D plot fitted to the fracture frequency as a kafirin concentration and internal frequency function. This indicates that increasing kafirin concentration (*K*; % [w/v]) and decreasing the value of internal frequency (*F*; Hz) resulted in the least breakage of the DDGS kafirin microparticles. Increasing the concentration of kafirin will allow it to form more intermolecular disulfide bonds and hence could be a possible reason for the increase in the mechanical strength of microparticles.

### 3.4 Morphological Analysis of Microparticles From Selected Modeling Runs

**FIGURE 4:**
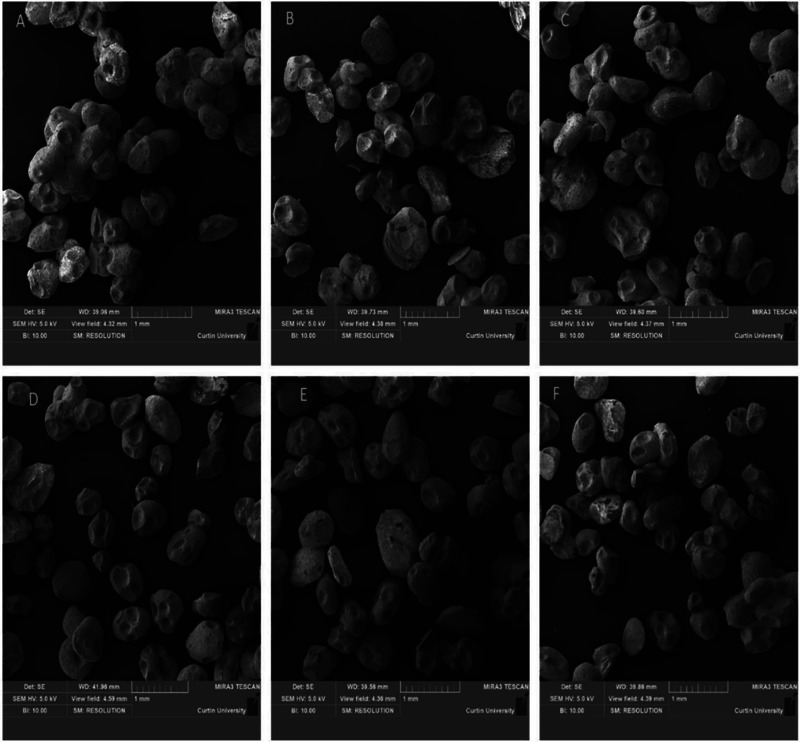
Field emission‐scanning electron micrograms (FE‐SEM) of DDGS kafirin microparticles at a magnification of 1000 µm and voltage of 5 kV using a secondary electron beam signal. The micrographs represent materials produced from modeling runs: (A) 1; (B) 5; (C) 9; (D) 12; (E) 16; and (F) 20.

SEM was used to investigate the integrity of the fabricated microparticles from six runs made with a wide range of process parameter levels (Figure 4A–F). The literature suggested that kafirin protein made from sorghum grain self‐assembles into spherical particles in aqueous systems (Taylor and Taylor 2018; Xiao et al. 2015), while DDGS kafirin was reported to form particles with nonuniform shapes (without sphericity) (Shah et al. 2021). This suggests that the high‐heat treatment used for the production of DDGS kafirin has caused heat‐induced disruption of internal bonds, leading to the re‐association of polypeptides into large microaggregates (Duodu et al. 2001; Gao et al. 2005; Shah et al. 2024; Shah et al. 2021). DDGS kafirin particles made using an evaporation‐induced self‐assembling method formed highly aggregated particles after being dried, and surface pores indicated a weak self‐assembling nature. In the present study using DDGS kafirin, microparticles formed were discrete microspheres with minimal aggregation and no pores on their surfaces (Figure 4A–F). However, the FE‐SEM images showed that microparticles made with a higher concentration of DDGS kafirin resulted in particle aggregation (Figure 4A,C).

The DDGS kafirin microparticles fabricated using the Run 1 condition (Table 2) were clumpy and linked together (Figure 4A). The DDGS kafirin microparticles produced under Run 9 (Figure 4B) resulted in the adhesion of particle surfaces with each other but less than those produced under Run 1 (Figure 4C). Thus, higher internal frequency in Run 9 (1600 Hz) manipulated DDGS kafirin microparticle morphology. The higher internal frequency might have led to the formation of an increased charge on the DDGS kafirin droplet. This higher charge leads to a uniform streamlined flow of DDGS kafirin microparticles (droplets) from the spray nozzle, reducing DDGS kafirin microdroplet collisions while solidifying in the gelation bath. These findings align with the zeta potential studies, as less negative surface charge was seen in the DDGS kafirin microparticles produced in Run 1 than in Run 9. The higher negative surface charge means less likelihood of DDGS kafirin microparticles forming clusters (Thielbeer et al., 2011). Therefore, IGVJFT is unsuitable for highly viscous DDGS kafirin mixtures because the morphological examination of microparticles produced from higher concentrations of DDGS kafirin indicated microparticle destabilization. Such findings are consistent with Coelho et al. (2021), who used spray drying to fabricate zein particles loaded with vitamin B12. The authors found that the delivery system (i.e., particles) had wrinkled surfaces at higher zein concentrations, and most microparticles were agglomerated.

The microparticle morphology prepared under conditions with a polymer concentration of ∼1% (w/v) has a stable shape, relatively smoother surfaces, and no particle agglomeration (Figure 4B,D,E). This can be attributed to the higher negative surface charge of the formulation at lower kafirin concentration. These findings align with the previous findings, suggesting that lower zein concentration produced better microparticle shape and size (Coelho et al. 2021).

### 3.5 Optimization of Modulated DDGS Kafirin Microparticles

The optimization aimed to search for a combination of process parameter levels that satisfy the target microparticle response. The goal was to achieve all of the following simultaneously: minimum volume‐weighted mean particle size (µm); as negative a zeta potential as possible (mV); and as low a fracture frequency as possible (%). The statistical solution with the highest desirability value, *D* = 1, was selected to optimize the responses. The optimal levels of the process parameter are 1.015% (w/v) kafirin concentration (rounded to 1% [w/v]), integrated voltage of 580.054 V (which was rounded to 580 V), and internal frequency of 1598 Hz. A statistical solution with a lower *D* value of 0.9 was selected as the suboptimal response. The suboptimal conditions are a kafirin concentration of 2% (w/v), an integrated electrode voltage of 200 V, and an internal frequency of 644.98 Hz (rounded to 645 Hz). Table 5 shows the model‐predicted and actual values of the dependent microparticle properties at the optimal and suboptimal conditions. The verification analysis demonstrated that the RSM model was able to predict values of all three microparticle properties since these response values were within the 95% prediction interval limits.

**TABLE 5:** Predicted and actual values for volume‐weighted mean microparticle size (PS; µm), zeta potential (ZP; mV), and fracture frequency (FF; %) of DDGS kafirin microparticles under optimal and suboptimal conditions.

Response	Optimal value		Suboptimal	
	Predicted	Actual	Predicted	Actual
PS (µm)	405 ± 12.7	409 ± 7.4	646 ± 32.4	654 ± 18.5
ZP (mV)	−38.2 ± 3.3	−35.9 ± 2.7	−16.7 ± 0.6	−19.6 ± 0.5
FF (%)	50 ± 5.1	47 ± 4.7	19 ± 0.7	20 ± 2

*Note*: All actual values were within the 95%.

## 4 Conclusion

The study successfully optimized the processing parameters of a newly established technology, IGVJFT, for the preparation of DDGS kafirin microparticles with desired physicochemical properties. This is the first report on the use of kafirin in microparticle assembly using IGVJFT, and as such, it demonstrates the feasibility of the technology for kafirin microparticle manufacture. The RSM‐based desirability function statistical modeling approach identified the optimal conditions for kafirin microparticle production using IGVJFT as an internal frequency of 1598 Hz, an integrated electrode voltage of 580 V, and a DDGS kafirin concentration of 1% (w/v). Under these conditions, the resulting microparticles exhibited an approximate volume‐weighted mean microparticle size of 406.7 µm, a highly negative zeta potential of −38.2 mV, and a low fracture frequency of 23%, demonstrating good colloidal stability and mechanical strength. Scanning electron microscopy validated the formation of spherical‐shaped microparticles. These findings provide a strong foundation for applying DDGS kafirin in biomaterial production, further showing the potential of IGVJFT as a novel and efficient technology for producing hydrophobic protein‐based microparticles with tunable physicochemical properties.

## Author Contributions

**Umar Shah**: methodology, software, data curation, investigation, validation, formal analysis, writing–original draft. **Rewati R. Bhattarai**: writing–review and editing, resources **Hani Al Salami**: writing–review and editing. **Chris Blanchard**: writing–review and editing, funding acquisition. **Stuart K. Johnson**: conceptualization, supervision, funding acquisition, project administration, writing–review and editing, resources.

## Conflicts of Interest

The authors declare no conflicts of interest.

## Supporting information



Suppl. Figure 1. One‐factor plot of volume‐weighted mean particle size of DDGS kafirin microparticles as a function of the concentration of kafirin (% w/v).

Suppl. Figure 2. One‐factor plot of zeta potential, ZP of DDGS kafirin microparticles as a function of kafirin concentration (% w/v).

**Suppl. Figure 3**. One‐factor plot of volume‐weighted mean of fracture frequency, FF as a function of kafirin concentration (% w/v).

Suppl. Figure 4. Diagnostics graphs of the selected model for the volume‐weighted mean of DDGS kafirin microparticle size: (A) normal probability plot (B) predicted plot.

Suppl. Figure 5. Diagnostics graphs of the selected model for zeta potential of DDGS kafirin microparticles (A) normal probability plot (B) predicted plot.

Suppl. Figure 6. Diagnostics graphs of the selected model for fracture frequency of DDGS kafirin microparticles (A) normal probability plot (B) predicted plot.

## References

[jfds70268-bib-0001] Belton, P. S. , I. Delgadillo , N. G. Halford , and P. R. Shewry . 2006. “Kafirin Structure and Functionality.” Journal of Cereal Science 44, no. 3: 272–286. 10.1016/j.jcs.2006.05.004.

[jfds70268-bib-0002] Chatzifragkou, A. , O. Kosik , P. C. Prabhakumari , et al. 2015. “Biorefinery Strategies for Upgrading Distillers' Dried Grains With Solubles (DDGS).” Process Biochemistry 50, no. 12: 2194–2207. 10.1016/j.procbio.2015.09.005.

[jfds70268-bib-0003] Coelho, S. C. , S. Laget , P. Benaut , F. Rocha , and B. N. Estevinho . 2021. “A New Approach to the Production of Zein Microstructures With Vitamin B12, by Electrospinning and Spray Drying Techniques.” Powder Technology 392: 47–57. 10.1016/j.powtec.2021.06.056.

[jfds70268-bib-0004] Davila, S. , C. Pérez‐García , and A. Feregrino‐Perez . 2021. “Challenges and Advantages of Electrospun Nanofibers in Agriculture: A Review.” Materials Research Express 8, no. 4: 042001. 10.1088/2053-1591/abee55.

[jfds70268-bib-0005] Duodu, K. G. , H. Tang , A. Grant , N. Wellner , P. S. Belton , and J. R. N. Taylor . 2001. “FTIR and Solid State^13^C NMR Spectroscopy of Proteins of Wet Cooked and Popped Sorghum and Maize.” Journal of Cereal Science 33, no. 3: 261–269. 10.1006/jcrs.2000.0352.

[jfds70268-bib-0006] Duodu, K. G. , J. R. N. Taylor , P. S. Belton , and B. R. Hamaker . 2003. “Factors Affecting Sorghum Protein Digestibility.” Journal of Cereal Science 38, no. 2: 117–131. 10.1016/S0733-5210(03)00016-X.

[jfds70268-bib-0007] Frank, J. R. 1992. “Experimental Design in Biotechnology, Statistics: Textbooks and Monographs, Volume 105 Perry D. Haaland Marcel Dekker, Inc., New York, NY, 259 Pages [ISBN No.: 0‐8247‐7881‐2]” Environmental Progress 11, no. 3: A8–A9. 10.1002/ep.670110307.

[jfds70268-bib-0008] Gao, C. , J. Taylor , N. Wellner , et al. 2005. “Effect of Preparation Conditions on Protein Secondary Structure and Biofilm Formation of Kafirin.” Journal of Agricultural and Food Chemistry 53: 306–312. 10.1021/jf0492666. 15656666

[jfds70268-bib-0009] Guazzelli, N. , L. Cacopardo , A. Corti , and A. Ahluwalia . 2023. “An Integrated In Silico‐In Vitro Approach for Bioprinting Core‐Shell Bioarchitectures.” International Journal of Bioprinting 9, no. 5: 771. 10.18063/ijb.771. 37457929 PMC10339450

[jfds70268-bib-0010] Han, J. , P. Wang , Y. Guo , et al. 2025. “Thermo‐Responsive Jamming by Particle Shape Change.” Nature Communications 16, no. 1: 2303. 10.1038/s41467-025-57475-5. PMC11889120 40055334

[jfds70268-bib-0011] Hill, R. J. , D. A. Saville , and W. B. Russel . 2003. “Electrophoresis of Spherical Polymer‐Coated Colloidal Particles.” Journal of Colloid and Interface Science 258, no. 1: 56–74. 10.1016/S0021-9797(02)00043-7. 14611793

[jfds70268-bib-0012] Hu, B. , Y. Yang , L. Han , J. Yang , W. Zheng , and J. Cao . 2022. “Characterization of Hydrophilic and Hydrophobic Core‐Shell Microcapsules Prepared Using a Range of Antisolvent Approaches.” Food Hydrocolloids 131: 107750. 10.1016/j.foodhyd.2022.107750.

[jfds70268-bib-0013] Jones, M. , D. Walker , C. M. Ionescu , et al. 2020. “Microencapsulation of Coenzyme Q10 and Bile Acids Using Ionic Gelation Vibrational Jet Flow Technology for Oral Delivery.” Therapeutic Delivery 11, no. 12: 791–805. 10.4155/tde-2020-0082. 33225829

[jfds70268-bib-0014] Kesimer, M. , and R. Gupta . 2015. “Physical Characterization and Profiling of Airway Epithelial Derived Exosomes Using Light Scattering.” Methods 87: 59–63. 10.1016/j.ymeth.2015.03.013. 25823850 PMC4584172

[jfds70268-bib-0015] Kour, P. , A. Shaheen , U. N. Tak , A. Gani , H. K. Qadri , and A. A. Dar . 2024. “Pickering Emulsions of Zein Nanoparticles Co‐Stabilized by Tween 20: An Effective Strategy to Stabilize Citral in Low pH Environment.” Colloids and Surfaces A: Physicochemical and Engineering Aspects 701: 134876. 10.1016/j.colsurfa.2024.134876.

[jfds70268-bib-0016] Kovacevic, B. , C. M. Ionescu , M. Jones , et al. 2024. “Novel Polysaccharides–Bile Acid–Cyclodextrin Gel Systems and Effects on Cellular Viability and Bioenergetic Parameters.” Therapeutic Delivery 15, no. 2: 119–134. 10.4155/tde-2023-0063. 38180012

[jfds70268-bib-0017] Lai, K. K. , R. Renneberg , and W. C. Mak . 2016. “High Efficiency Single‐Step Biomaterial‐Based Microparticle Fabrication via Template‐Directed Supramolecular Coordination Chemistry.” Green Chemistry 18, no. 6: 1715–1723. 10.1039/C5GC02424B.

[jfds70268-bib-0018] Lau, E. T. L. , S. K. Johnson , R. A. Stanley , et al. 2015. “Formulation and Characterization of Drug‐Loaded Microparticles Using Distillers Dried Grain Kafirin.” Cereal Chemistry 92, no. 3: 246–252. 10.1094/CCHEM-05-14-0096-R.

[jfds70268-bib-0019] Lee, S. , D. Heng , W. Ng , H.‐K. Chan , and R. Tan . 2010. “Nano Spray Drying: A Novel Method for Preparing Protein Nanoparticles for Protein Therapy.” International Journal of Pharmaceutics 403: 192–200. 10.1016/j.ijpharm.2010.10.012. 20951781

[jfds70268-bib-0020] Leong, T. S. H. , G. J. O. Martin , and M. Ashokkumar . 2017. “Ultrasonic Encapsulation—A Review.” Ultrasonics Sonochemistry 35: 605–614. 10.1016/j.ultsonch.2016.03.017. 27053430

[jfds70268-bib-0021] Lin, T. Y. , and S. N. Timasheff . 1996. “On the Role of Surface Tension in the Stabilization of Globular Proteins.” Protein Science 5, no. 2: 372–381. 10.1002/pro.5560050222. 8745416 PMC2143343

[jfds70268-bib-0022] Liu, G. , D. Wei , H. Wang , Y. Hu , and Y. Jiang . 2016. “Self‐Assembly of Zein Microspheres With Controllable Particle Size and Narrow Distribution Using a Novel Built‐In Ultrasonic Dialysis Process.” Chemical Engineering Journal 284: 1094–1105. 10.1016/j.cej.2015.09.067.

[jfds70268-bib-0023] Lowry, G. V. , R. J. Hill , S. Harper , et al. 2016. “Guidance to Improve the Scientific Value of Zeta‐Potential Measurements in NanoEHS.” Environmental Science: Nano 3, no. 5: 953–965. 10.1039/C6EN00136J.

[jfds70268-bib-0024] Mooranian, A. , R. Negrulj , R. Takechi , E. Jamieson , G. Morahan , and H. Al‐Salami . 2018. “Influence of Biotechnological Processes, Speed of Formulation Flow and Cellular Concurrent Stream‐Integration on Insulin Production From β‐Cells as a Result of Co‐Encapsulation With a Highly Lipophilic Bile Acid.” Cellular and Molecular Bioengineering 11, no. 1: 65–75. 10.1007/s12195-017-0510-y. 31719879 PMC6816685

[jfds70268-bib-0025] Mooranian, A. , S. Raj Wagle , B. Kovacevic , et al. 2020. “Bile Acid Bio‐Nanoencapsulation Improved Drug Targeted‐Delivery and Pharmacological Effects via Cellular Flux: 6‐Months Diabetes Preclinical Study.” Scientific Reports 10, no. 1: 106. 10.1038/s41598-019-53999-1. 31919411 PMC6952395

[jfds70268-bib-0026] Mushtaq, M. , A. Ayoub , J. A. Banday , A. Rashid , N. Rasheed , and A. Gani . 2025. “Nano‐Reduction of Whey Protein Using Ultra‐Sonication as a Novel Approach to Improve Its Applicability in Food Industry.” Ultrasonics Sonochemistry 114: 107230. 10.1016/j.ultsonch.2025.107230. 39954361 PMC11872402

[jfds70268-bib-0027] Nazir, M. , F. Jhan , A. Gani , and A. Gani . 2024. “Fabrication of Millet Starch Nanocapsules Loaded With Beta Carotene Using Acid Hydrolysis and Ultrasonication: Characterisation, Release Behaviour and Bioactivity Retention.” Ultrasonics Sonochemistry 111: 107112. 10.1016/j.ultsonch.2024.107112. 39447532 PMC11539498

[jfds70268-bib-0028] Patel, A. , A. Mohanan , and S. Ghosh . 2019. “Effect of Protein Type, Concentration and Oil Droplet Size on the Formation of Repulsively Jammed Elastic Nanoemulsion Gels.” Soft Matter 15, no. 47: 9762–9775. 10.1039/C9SM01650C. 31742298

[jfds70268-bib-0029] Quispe‐Condori, S. , M. D. A. Saldaña , and F. Temelli . 2011. “Microencapsulation of Flax Oil With Zein Using Spray and Freeze Drying.” LWT – Food Science and Technology 44, no. 9: 1880–1887. 10.1016/j.lwt.2011.01.005.

[jfds70268-bib-0030] Rosenboom, J.‐G. , R. Langer , and G. Traverso . 2022. “Bioplastics for a Circular Economy.” Nature Reviews Materials 7, no. 2: 117–137. 10.1038/s41578-021-00407-8. 35075395 PMC8771173

[jfds70268-bib-0031] Rostamabadi, H. , E. Assadpour , H. S. Tabarestani , S. R. Falsafi , and S. M. Jafari . 2020. “Electrospinning Approach for Nanoencapsulation of Bioactive Compounds; Recent Advances and Innovations.” Trends in Food Science & Technology 100: 190–209. 10.1016/j.tifs.2020.04.012.

[jfds70268-bib-0032] Semwal, J. , and M. S. Meera . 2024. “Novel Mode of Kafirin Modification Using Combination of Enzyme and Thermal Treatment to Expand Its Food Application.” Food Chemistry 460: 140489. 10.1016/j.foodchem.2024.140489. 39047474

[jfds70268-bib-0033] Shah, U. , R. Bhattarai , H. Al‐Salami , C. Blanchard , and S. K. Johnson . 2024. “Advances in Extraction, Structure, and Physiochemical Properties of Sorghum Kafirin for Biomaterial Applications: A Review.” Journal of Functional Biomaterials 15, no. 7: 172. 10.3390/jfb15070172. 39057294 PMC11278494

[jfds70268-bib-0034] Shah, U. , D. Dwivedi , M. Hackett , et al. 2021. “Physicochemical Characterisation of Kafirins Extracted From Sorghum Grain and Dried Distillers Grain With Solubles Related to Their Biomaterial Functionality.” Scientific Reports 11, no. 1: 15204. 10.1038/s41598-021-94718-z. 34312467 PMC8313537

[jfds70268-bib-0035] Shukla, R. , and M. Cheryan . 2001. “Zein: The Industrial Protein From Corn.” Industrial Crops and Products 13, no. 3: 171–192. 10.1016/S0926-6690(00)00064-9.

[jfds70268-bib-0036] Sincari, V. , S. Lukáš Petrova , E. Jäger , et al. 2024. “pH‐Induced Morphological Reversible Transition From Microparticles to Vesicles for Effective Bacteria Entrapment.” European Polymer Journal 221: 113511. 10.1016/j.eurpolymj.2024.113511.

[jfds70268-bib-0037] Song, J. , C. Sun , K. Gul , A. Mata , and Y. Fang . 2021. “Prolamin‐Based Complexes: Structure Design and Food‐Related Applications.” Comprehensive Reviews in Food Science and Food Safety 20, no. 2: 1120–1149. 10.1111/1541-4337.12713. 33569884

[jfds70268-bib-0038] Sun, M. , W. Tan , Z. Pang , C. Chen , H. Li , and X. Liu . 2025. “Rheological and Tribological Characteristics of Soy‐Whey Dual‐Protein Gels With Whey Protein Microparticle Incorporation.” Food Research International 208: 116167. 10.1016/j.foodres.2025.116167. 40263831

[jfds70268-bib-0039] Taylor, J. , and J. R. N. Taylor . 2018. “Making Kafirin, the Sorghum Prolamin, Into a Viable Alternative Protein Source.” Journal of the American Oil Chemists' Society 95, no. 8: 969–990. 10.1002/aocs.12016.

[jfds70268-bib-0040] Taylor, J. , J. R. N. Taylor , P. S. Belton , and A. Minnaar . 2009b. “Formation of Kafirin Microparticles by Phase Separation From an Organic Acid and Their Characterisation.” Journal of Cereal Science 50, no. 1: 99–105. 10.1016/j.jcs.2009.03.005.

[jfds70268-bib-0055] Thielbeer, F. , K. Donaldson , and M. Bradley . 2011. “Zeta Potential Mediated Reaction Monitoring on Nano and Microparticles.” Bioconjugate chemistry 22, no. 2: 144–150. 10.1021/bc1005015. 21244000

[jfds70268-bib-0041] Vera Candioti, L. , M. M. De Zan , M. S. Cámara , and H. C. Goicoechea . 2014. “Experimental Design and Multiple Response Optimization. Using the Desirability Function in Analytical Methods Development.” Talanta 124: 123–138. 10.1016/j.talanta.2014.01.034. 24767454

[jfds70268-bib-0042] Verma, R. , and K. S. Daya . 2017. “Understanding the Decay of Proteins: A Method to Study Time Dependent Response of pM Concentration of Insulin at Microwave Frequencies.” MethodsX 4: 35–41. 10.1016/j.mex.2016.11.004. 28116247 PMC5233787

[jfds70268-bib-0043] Villarino, B. , V. Jayasena , R. Coorey , S. Chakrabarti‐Bell , and S. Johnson . 2015. “Optimization of Formulation and Process of Australian Sweet Lupin (ASL)‐Wheat Bread.” LWT – Food Science and Technology 61, no. 2: 359–367. 10.1016/j.lwt.2014.11.029.

[jfds70268-bib-0044] Vladisavljević, G. T. 2024. “Preparation of Microparticles and Nanoparticles Using Membrane‐Assisted Dispersion, Micromixing, and Evaporation Processes.” Particuology 84: 30–44. 10.1016/j.partic.2023.03.003.

[jfds70268-bib-0045] Vossoughi, A. , and H. W. T. Matthew . 2018. “Encapsulation of Mesenchymal Stem Cells in Glycosaminoglycans‐Chitosan Polyelectrolyte Microcapsules Using Electrospraying Technique: Investigating Capsule Morphology and Cell Viability.” Bioengineering & Translational Medicine 3, no. 3: 265–274. 10.1002/btm2.10111. 30377665 PMC6195902

[jfds70268-bib-0046] Wagle, S. R. , B. Kovacevic , T. Foster , et al. 2024. “Probucol‐Bile Acid Nanoparticles: A Novel Approach and Promising Solution to Prevent Cellular Oxidative Stress in Sensorineural Hearing Loss.” Journal of Drug Targeting 32, no. 7: 737–755. 10.1080/1061186X.2024.2349111. 38758361

[jfds70268-bib-0047] Wagle, S. R. , B. Kovacevic , D. Walker , et al. 2020. “Alginate‐Based Drug Oral Targeting Using Bio‐Micro/Nano Encapsulation Technologies.” Expert Opinion on Drug Delivery 17, no. 10: 1361–1376. 10.1080/17425247.2020.1789587. 32597249

[jfds70268-bib-0048] Wang, Y. , M. Tilley , S. Bean , S. X. Sun , and D. Wang . 2009. “Comparison of Methods for Extracting Kafirin Proteins From Sorghum Distillers Dried Grains With Solubles.” Journal of Agricultural and Food Chemistry 57: 8366–8372. 10.1021/jf901713w. 19754169

[jfds70268-bib-0049] Xiao, J. , Y. Chen , and Q. Huang . 2017. “Physicochemical Properties of Kafirin Protein and Its Applications as Building Blocks of Functional Delivery Systems.” Food & Function 8, no. 4: 1402–1413. 10.1039/C6FO01217E. 28256656

[jfds70268-bib-0050] Xiao, J. , Y. Li , J. Li , A. P. Gonzalez , Q. Xia , and Q. Huang . 2015. “Structure, Morphology, and Assembly Behavior of kafirin.” Journal of Agricultural and Food Chemistry 63, no. 1: 216–224. 10.1021/jf504674z. 25510968 PMC4298357

[jfds70268-bib-0051] Xiao, J. , X. Wang , A. Perez Gonzalez , and Q. Huang . 2016. “Kafirin Nanoparticles‐Stabilized Pickering Emulsions: Microstructure and Rheological Behavior.” Food Hydrocolloids 54: 30–39. 10.1016/j.foodhyd.2015.09.008.

[jfds70268-bib-0052] Xu, L. , J.‐J. Li , D.‐G. Yu , G. R. Williams , J.‐H. Yang , and X. Wang . 2017. “Influence of the Drug Distribution in Electrospun Gliadin Fibers on Drug‐Release Behavior.” European Journal of Pharmaceutical Science 106: 422–430. 10.1016/j.ejps.2017.06.017. 28614732

[jfds70268-bib-0053] Ye, Z. , Y. Wang , P. Shen , L. M. C. Sagis , and J. Landman . 2024. “Effect of Gum Arabic Coating on Release Behavior of Curcumin‐Loaded Kafirin and Zein Composite Nanoparticles.” Food Hydrocolloids 156: 110254. 10.1016/j.foodhyd.2024.110254.

[jfds70268-bib-0054] Zhong, L. , Z. Fang , M. L. Wahlqvist , J. M. Hodgson , and S. K. Johnson . 2019. “Extrusion Cooking Increases Soluble Dietary Fibre of Lupin Seed Coat.” LWT – Food Science and Technology 99: 547–554. 10.1016/j.lwt.2018.10.018.

