# Supplementary material for: Multiresponse Surface Optimization of Ionic Gelation Vibrational Jet Flow Technology to Fine‐Tune Kafirin Microparticles Extracted From Sorghum Dried Distiller's Grain
Source: J Food Sci. 2025 May 22;90(5):e70268. doi: 10.1111/1750-3841.70268 (PMC12096266; doi:10.1111/1750-3841.70268)
Supplement: Supplementary file 1 — Suppl. Figure 1. One‐factor plot of volume‐weighted mean particle size of DDGS kafirin microparticles as a function of the concentration of kafirin (% w/v). [file JFDS-90-0-s001.docx]

**Multi-response surface optimisation of ionic gelation vibrational jet flow technology to fine-tune kafirin microparticles extracted from sorghum dried distiller’s grain**

Umar Shah^1^ [umarshah.umarzahoor@gmail.com](mailto:umarshah.umarzahoor@gmail.com)

Rewati Bhattarai^1^ [r.bhattarai@curtin.edu.au](mailto:r.bhattarai@curtin.edu.au)

Hani Al Salami^3,^ [hani.alsalami@curtin.edu.au](mailto:hani.alsalami@curtin.edu.au)

Chris Blanchard^2^ [cblanchard@csu.edu.au](mailto:cblanchard@csu.edu.au)

Stuart K. Johnson^1^ [stuart@ingredientsbydesign.com.au](mailto:stuart@ingredientsbydesign.com.au)

^1^School of Molecular and Life Sciences, Faculty of Science and Engineering, Curtin University, GPO Box U1987, Perth, WA 6845, Australia.

^2^ARC ITTC for Functional Grains, Graham Centre for Agricultural Innovation, Charles Sturt University, Wagga Wagga, NSW, 2678, Australia.

^3^The Biotechnology and Drug Development Research Laboratory, Curtin Medical School and Curtin Health Innovation Research Institute, Curtin University, GPO Box U1987, Perth WA, 6845, Australia

**Correspondence**: Dr Stuart K. Johnson

Email: [stuart@ingredientsbydesign.com.au](mailto:stuart@ingredientsbydesign.com.au)

+61 (0) 410781873

**Supplementary information**

**Multi-response surface optimisation of ionic gelation vibrational jet flow technology to fine-tune kafirin microparticles extracted from sorghum dried distillers grain**

Statistical data analysis central composite design of response surface methodology

**Suppl. Figure 1.** One-factor plot of volume-weighted mean particle size of DDGS kafirin microparticles as a function of the concentration of kafirin (% w/v).

**Suppl. Figure 2.** One-factor plot of zeta potential, ZP of DDGS kafirin microparticles as a function of kafirin concentration (% w/v).

**Suppl. Figure 3.** One-factor plot of volume-weighted mean of fracture frequency, FF as a function of kafirin concentration (% w/v).


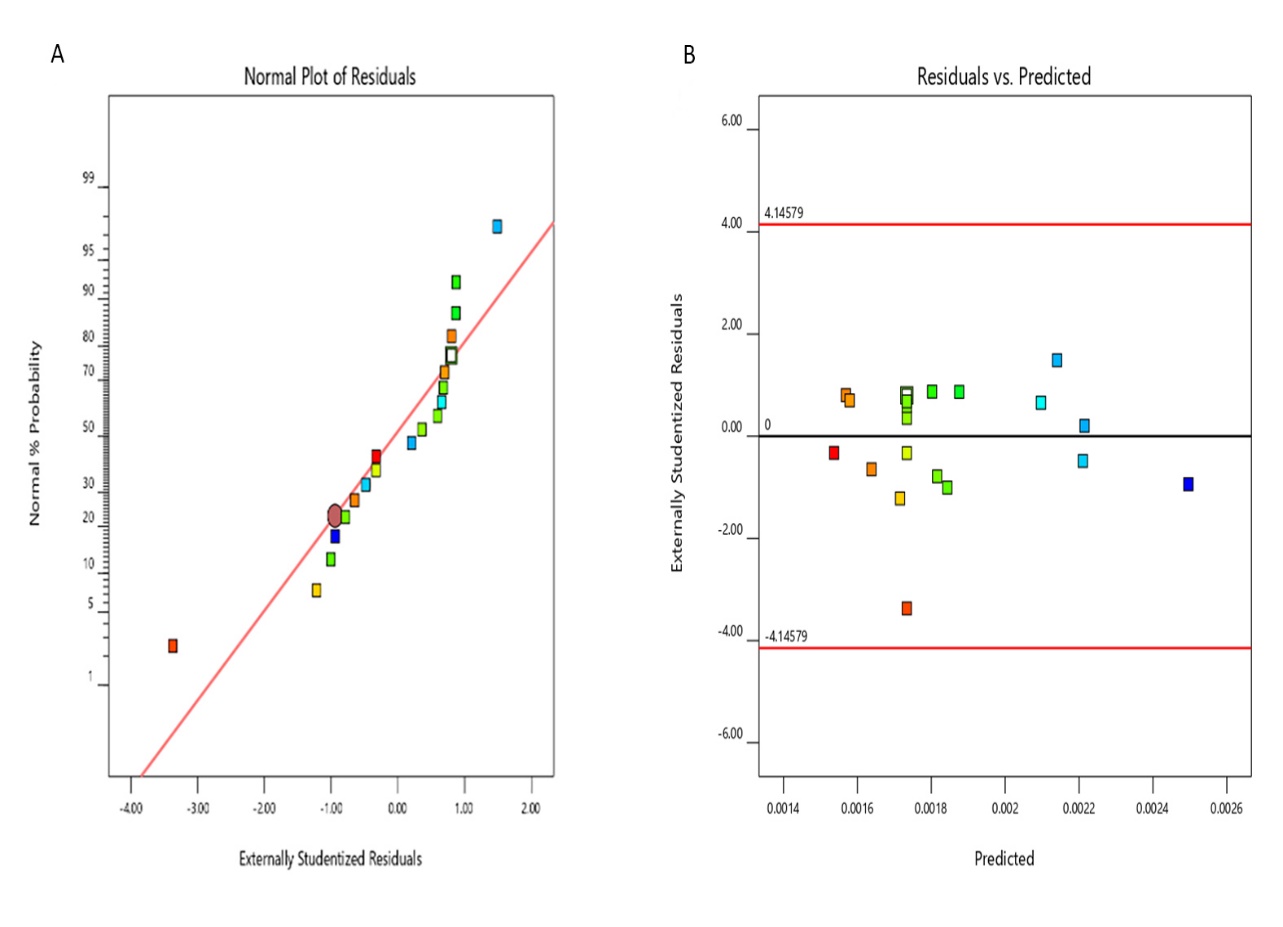


**Suppl. Figure 5.4.** Diagnostics graphs of the selected model for the volume-weighted mean of DDGS kafirin microparticle size: (A) normal probability plot (B) predicted plot.


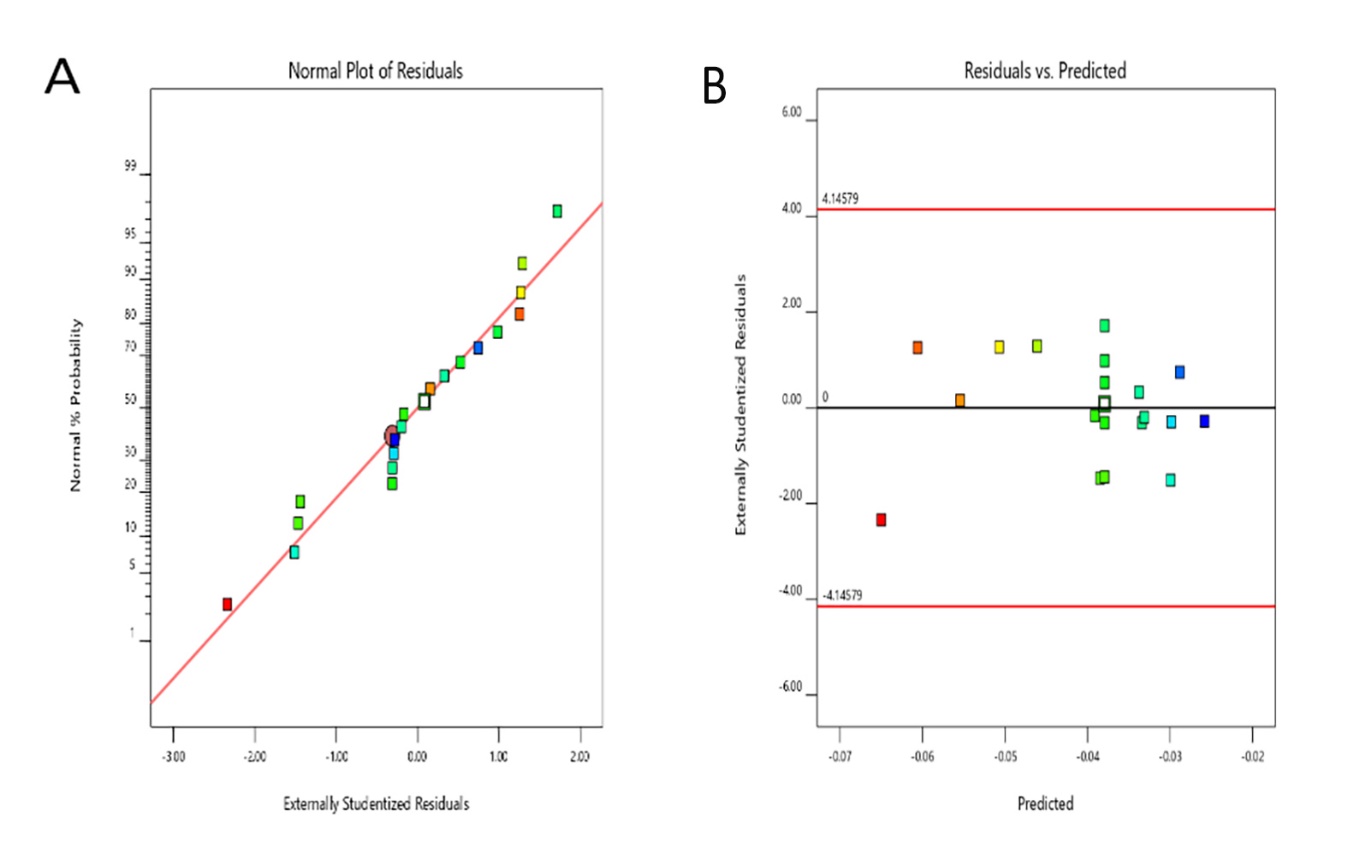


**Suppl. Figure 5.** Diagnostics graphs of the selected model for zeta potential of DDGS kafirin microparticles (A) normal probability plot (B) predicted plot.


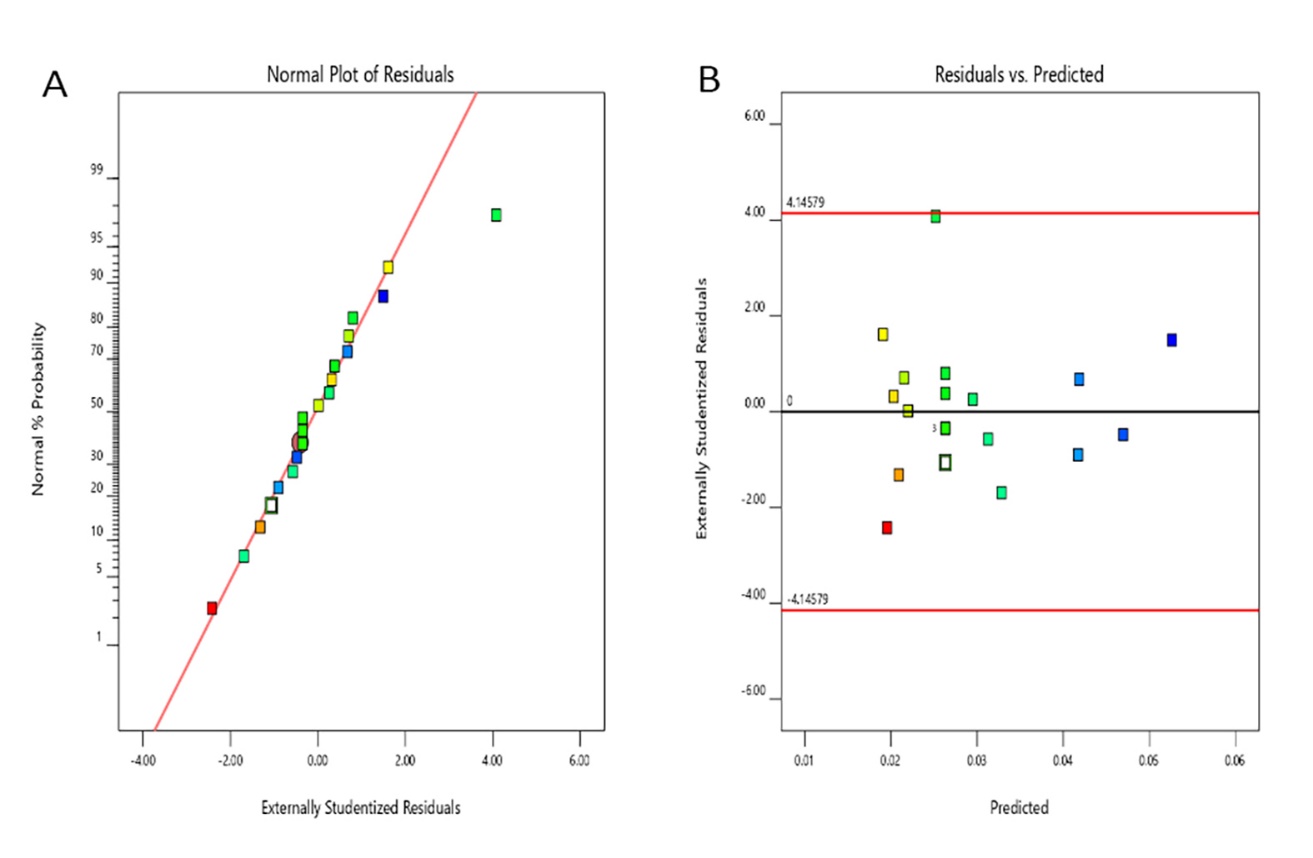


**Suppl. Figure 6.** Diagnostics graphs of the selected model for fracture frequency of DDGS kafirin microparticles (A) normal probability plot (B) predicted plot.
